# Programmed Death-1/Programmed Death-Ligand 1-Axis Blockade in Recurrent or Metastatic Head and Neck Squamous Cell Carcinoma Stratified by Human Papillomavirus Status: A Systematic Review and Meta-Analysis

**DOI:** 10.3389/fimmu.2021.645170

**Published:** 2021-04-07

**Authors:** Yimin Xu, Gangcai Zhu, Christopher A. Maroun, Irene X. Y. Wu, Donghai Huang, Tanguy Y. Seiwert, Yong Liu, Rajarsi Mandal, Xin Zhang

**Affiliations:** ^1^ Department of Otolaryngology-Head and Neck Surgery, Xiangya Hospital, Central South University, Changsha, China; ^2^ Clinical Research Center for Pharyngolaryngeal Diseases and Voice Disorders in Hunan Province, Changsha, China; ^3^ Department of Otolaryngology-Head and Neck Surgery, The Second Xiangya Hospital, Central South University, Changsha, China; ^4^ Department of Otolaryngology-Head and Neck Surgery, Johns Hopkins University, Baltimore, MD, United States; ^5^ Bloomberg–Kimmel Institute for Cancer Immunotherapy at Johns Hopkins Medicine, Baltimore, MD, United States; ^6^ Department of Epidemiology and Health Statistics, Xiangya School of Public Health, Central South University, Changsha, China; ^7^ National Clinical Research Center for Geriatric Disorders, XiangYa Hospital, Changsha, China; ^8^ Department of Oncology, Johns Hopkins University, Baltimore, MD, United States; ^9^ Otolaryngology Major Disease Research Key Laboratory of Hunan Province, Changsha, China

**Keywords:** human papilloma virus, immune checkpoint blockade, head and neck squamous cell carcinoma, anti-PD-1, anti-PD-L1

## Abstract

**Background:**

Programmed death-1/programmed death-ligand 1 (PD-1/PD-L1) inhibitors have provided clinical benefit to head and neck squamous cell carcinoma (HNSCC) patients in recent clinical trials. However, it remains unclear as to whether human papillomavirus (HPV) status is associated with improved clinical outcome of anti-PD-1 or anti-PD-L1 immunotherapy in HNSCC.

**Methods:**

PubMed, EMBASE, Cochrane Library, and Web of Science were systematically searched up to February 28, 2021. Published clinical trials of HNSCC patients treated with only PD-1 or PD-L1 inhibitors were selected. The primary or secondary outcome of these studies included objective response rate (ORR) stratified by HPV status. The pooled odds ratio (OR) and hazard ratio (HR) were estimated using a fixed-effect model.

**Results:**

A total of seven eligible studies comprising 814 patients were included. The ORR of HPV positive HNSCC patients was significantly higher than that of HPV negative HNSCC patients (OR = 1.77; 95%CI = 1.14-2.74; *P* = 0.01), and this favorable effect occurred in pooled anti-PD-L1 trials (OR = 2.66; 95%CI = 1.16-6.11; *P* = 0.02). In comparison, the pooled OR was 1.51 in anti-PD-1 trials (95%CI = 0.90-2.54; *P* = 0.12). Survival analysis indicated that HPV positive HNSCC patients had a lower risk of overall death as compared to HPV negative HNSCC patients (HR = 0.77; 95%CI = 0.60–0.99; *P* = 0.04).

**Conclusions:**

HPV positive HNSCC patients display improved outcomes with PD-1/PD-L1 axis blockade as compared to HPV negative HNSCC patients. These improved outcomes are likely driven to a greater extent by anti-PD-L1 inhibitors. However, randomized controlled trials with greater numbers of patients are needed for validation of these early findings.

## Introduction

Head and neck squamous cell carcinoma (HNSCC) is the sixth most common cancer globally, with 600,000 cases diagnosed annually and mortality rates as high as 40%–50% (1). The vast majority of head and neck cancers are squamous cell carcinomas, which arise within different anatomical subsites. Therapeutic strategies for HNSCC include surgery, chemotherapy, radiotherapy, and targeted agents, including small molecular inhibitors or antibodies (2). Despite advances in treatment, the estimated 5-year overall survival rate of HNSCC has not significantly improved (3). Recently, there have been several studies that show immune checkpoint blockade appears to provide a promising new avenue for treatment in HNSCC (4, 5).

Programmed death-1 (PD-1), a member of the immunoglobulin superfamily associated with CD28 and CTLA-4, may be expressed on the surface of activated T cells, B cells, and monocytes (6). Programmed death-ligand 1 (PD-L1) binding to PD-1 on T cells results in suppression of the T cell immune response (7). Cancer cells may develop several mechanisms of escaping immune-mediated surveillance and death, including surface expression of PD-L1 (8). The interruption of PD-1 engagement by its ligand reinvigorates the immune system, allowing immune-mediated anti-cancer responses to resume, leading to marked clinical responses in some cancers (9). Anti-PD-1 and anti-PD-L1 antibodies such as nivolumab, pembrolizumab, cemiplimab, and atezolizumab, durvalumab, avelumab have shown promising results in several cancer types (10, 11). Nivolumab and pembrolizumab have been approved as first-line agents in recurrent/metastatic HNSCC patients by the United States Food and Drug Administration (FDA) (7, 12).

Despite a declining trend in smoking and drinking rates in the United States, the incidence of a proportion of HNSCC related to human papillomavirus (HPV) infection has been increasing (13). HPV positive and negative HNSCC are considered two entirely different types of cancer, in part due to their unique molecular landscapes (14). HPV associated oncogenes E6 and E7 drive oncogenesis in HNSCC by inactivating tumor suppressors TP53 and Rb and activating oncogenic signaling pathways including EGFR and PI3K etc (15, 16). Nevertheless, numerous clinical studies have demonstrated that HPV positivity in HNSCC confers a clear survival benefit as compared to HPV negative HNSCC patients after surgery with or without chemoradiotherapy (17, 18). One possible factor contributing to this survival difference is that HPV may elicit inherent local or systemic immunity against tumor cells in HNSCC patients, even in the absence of therapy (19), leading to the hypothesis that HPV positive HNSCC patients may show increased benefit from immune checkpoint blockade.

There have been conflicting results from published HNSCC clinical trials involving either anti-PD-1 or anti-PD-L1 therapy. The HAWK (20) study concluded that HPV positive patients had a higher objective response rate and survival rate than HPV negative patients. However, Keynote012 (21), NCT01375842 (22), and Keynote055 (23) trials reported that HNSCC patients’ tumor response did not correlate with HPV status. Data from two recent meta-analyses (24, 25) suggest there is a trend towards significance favoring higher response rates in HPV positive vs. HPV negative tumors in patients receiving anti-PD-1/PD-L1 therapy. One study by Wang et al. (25) used odds ratio (OR) in the analysis of overall survival, however this calculation does not take into account the effect of time. Furthermore, key limitations in these studies include a lack of stratification by anti-PD-1 or anti-PD-L1 therapy separately and inadequate selection of trials based on what is publicly available. Based on these inconsistent findings, we posited that there might be a difference in outcomes in HPV positive patients treated with immunotherapy depending on the use of either PD-1 or PD-L1 agents disrupting the PD-1/PD-L1 axis.

To further understand the importance of HPV status in HNSCC patients treated with anti-PD-1 or anti-PD-L1 agents, we systematically pooled the results from available trials together and conducted the present meta-analysis, which ultimately may help inform further investigation and ultimately clinical decision making.

## Materials and Methods

### Search Strategy and Eligibility Criteria

This systematic review and meta-analysis was conducted according to the Cochrane Handbook for Systematic Reviews of Interventions (26) and reported by adhering to the Preferred Reporting Items for Systematic Reviews and Meta-Analyses: the PRISMA Statement (27). Our protocol has been registered in the PROSPERO platform (ID: CRD42020175779).

Two independent authors systematically searched PubMed, EMBASE, Cochrane Library, and Web of Science for relevant articles published in English until February 28, 2021. Our search strategies included the following terms: “HPV or Human papillomavirus”, “Immunotherapy or Cemiplimab or Atezolizumab or Nivolumab or Pembrolizumab or Durvalumab or Avelumab or PD-1 or PD-L1 or PD1 or PDL1 or checkpoint” and “head and neck or head and neck cancer or head and neck neoplasm or head and neck tumor or head and neck carcinoma or HNC or HNSCC or SCCHN”. The complete search strategies used are found in **Supplementary Data**. We also manually checked the reference lists of identified studies and reviews to include more eligible trials. The search results were imported into Endnote (version 9.2).

Studies were included if they satisfied the following criteria: clinical trials of HNSCC patients treated with only a PD-1 or PD-L1 inhibitor agent, regardless of region, race, age, and gender; studies with a primary or secondary outcome that included objective response rate (ORR); reporting of ORR stratified by HPV status; studies reported in English. Clinical trials allowing participants with prior exposure to any immune-checkpoint blockade were excluded. If the same clinical study was reported in more than one publication, only the one with the most recent or complete data was analyzed. The methodological quality of randomized controlled trials (RCT) was evaluated by the recommendations in the Cochrane Collaboration handbook (26) to assess the risk of bias. The quality of non-RCTs was judged by the Newcastle-Ottawa Scale (28) by two of the authors independently.

### Data Extraction

We included the following data extracted from the eligible studies: trial name, publication year, study design, drug and dose, number of participants, age, gender, HPV status, anatomical subsite, ORR, overall survival (OS), progression-free survival (PFS), median time of follow-up, median OS, median PFS, median duration of response and the median time to response. The disease control rate (DCR) was extracted as the percentage of patients with complete response, partial response, or stable disease in the trial according to the guideline of response evaluation criteria in solid tumors (RECIST version 1.1) (29).

### Statistical Analysis

Continuous variables were reported as mean ± standard deviation (or median and range). Categorical variables were expressed as count and percentage. Measures of ORR and DCR stratified by HPV status were assessed by odds ratio (OR) and 95% confidence interval (CI). Subgroup analysis of ORR was performed according to the treatment agent used. OS and PFS data were evaluated by hazard ratio (HR) and 95% CI, and Tierney methodology was used for calculation if the data were not directly available in the original report (30). Statistical heterogeneity was detected using the Cochran Q chi-square test and inconsistency index (I^2^). If the studies were low heterogeneity (P>0.1, I^2^ < 50%), a fixed-effects model was used. Otherwise, a random-effects model was applied. We did not assess publication bias because only a small number of studies were included in the meta-analysis (n_max_ = 7). All statistical analyses were conducted with Review Manager version 5.3 and STATA version 16.

## Results

### Study Search, Selection, and Characteristics

**Figure 1:**
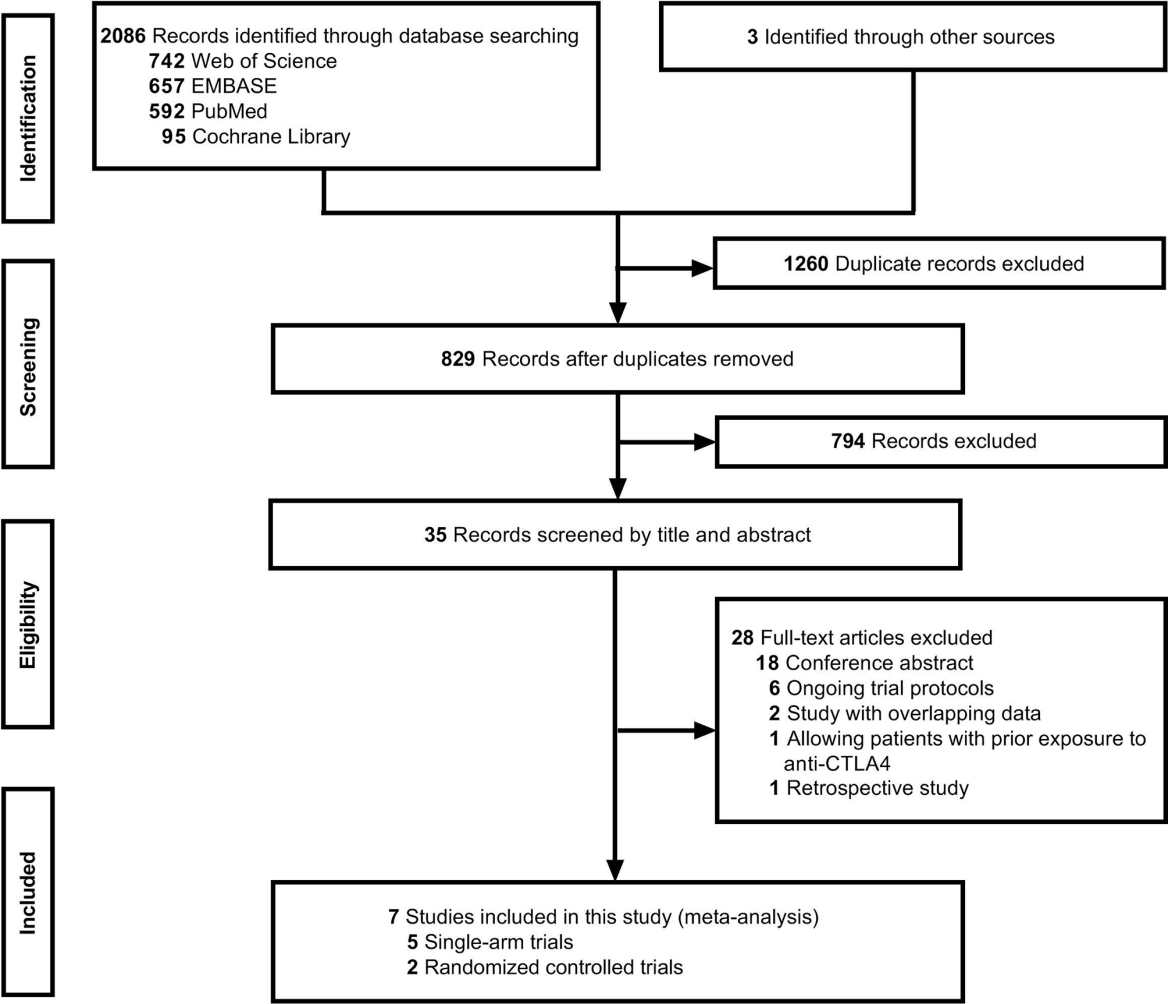
Study selection followed by PRISMA diagram.

A literature search identified 829 records after removing duplicates, and seven studies (20–23, 31–33) met the inclusion criteria after screening by title, abstract, and full text (**Figure 1**). The seven clinical trials included patients treated with anti-PD-1/PD-L1 agents and standard treatment and were comprised of two randomized controlled trials (RCT) and five single-arm trials.

The summary of the risk of bias for the two RCTs was shown in **Supplementary Figure 1**. The Newcastle-Ottowa Scale (28) score of the five single-arm studies was 5 (**Supplementary Table 1**).

### Patients Characteristics

A total of 814 patients were included, 671 (82.4%) of which had HPV status reported (**Table 1** **)**. Most of the patients were male (80.5%); the mean of the median age was 60.2 years (range 20-90) across the included trials. There were 217 (32.3%) HPV positive patients and 454 (67.7%) HPV negative patients. A summary of the anatomical subsites included was reported in **Supplementary Table 2**. The most common subsite in included trials was the oropharynx (n = 259, 31.8%). As shown in **Table 2**, the median OS and duration of response across the included studies were longer than the standard therapy arm in the Checkmate141 study (6.0-13.0 months vs. 5.1 months, 7.4-12.4 months vs. 4.0 months) (31).

**Table 1:** The characteristics of the included studies.

Study	Year	Study design(Open-label)	Drug and dose	N	Age(median, range)	Male(%)	HPV status			
							Method	+ (%)	- (%)	unknown (%)
Anti-PD-1										
Checkmate141 2y update	2018	Randomized, phase III	Nivolumab 3mg/kg, iv, every 2weeks	240	59 (29-83)	197 (82)	OPC used p16 IHC test, >70% is +	64 (27)	56 (23)	120 (50)
Keynote012	2016	Non-randomized, multicenter, multi-cohort, phase I b	Pembrolizumab 10mg/kg, iv, every 2 weeks	60	63 (20-83)	49 (82)	p16 IHC test, >70% is +	23 (38)	37 (62)	0 (0)
Keynote012 expansion	2016	Non-randomized, multicenter, multi-cohort, phase I b	Pembrolizumab 200mg, iv, every 3 weeks	132	60 (25-84)	110 (83)	the site investigator	28 (21)	104 (79)	0 (0)
Keynote055	2017	Multicenter, single-arm, phase II	Pembrolizumab 200 mg, iv, every 3 weeks	171	60 (33-90)	138 (81)	Local institution (most use p16 IHC test)	37 (22)	131 (77)	3 (1)
Anti-PD-L1										
HAWK	2019	Single-arm, phase II	Durvalumab 10 mg/kg, iv, every 2 weeks	112	60 (24-84)	80 (71)	p16 IHC test, FISH or PCR	34 (30)	65 (58)	13 (12)
CONDOR	2018	Randomized, multicenter, phase II	Durvalumab 10 mg/kg, iv, every 2 weeks	67	62 (23-82)	54 (81)	Medical records, local or central testing	18 (27)	49 (73)	0 (0)
NCT01375842	2018	Phase I a	Atezolizumab 15mg/kg, 20mg/kg, or a 1200-mg fixed dose, iv, every 3weeks	32^a^	62 (32-78)	27 (84)	PCR	13 (41)	12 (38)	3 (9)

N, number of patients; PD-1, programmed death 1; PD-L1, programmed death-ligand 1; HPV, human papillomavirus; +, positive; -, negative; iv, intravenous; OPC, oropharyngeal cancer; IHC, immunohistochemistry; FISH, fluorescence in situ hybridization; PCR, polymerase chain reaction.

a In NCT01375842, four patients with nasopharyngeal cancer were excluded from the HPV analysis population.

**Table 2:** The response and survival time of included studies.

Study	Drug	ORR(%)	DCR(%)	Median follow-up (months)	Median OS(months)	Median PFS(months)	Median duration of response (months)	Median time to response (months)
Anti-PD-1								
Checkmate141 2y update, 2018	Nivolumab	Niv: 13.3 IC^a^: 5.8	36.3 41.3	NA^b^	7.7 5.1	2.0 2.3	9.7 4.0	2.1 2.0
Keynote012, 2016	Pembrolizumab	21.4	48.2	14 (IQR, 4-14)	13.0	2.0	12.4	1.9
Keynote012 expansion, 2016	Pembrolizumab	17.7	34.9	9 (IQR, 3-11)	8.0	2.0	not reached	2.0
Keynote055, 2017	Pembrolizumab	16.4	35.7	7 (range, 0-17)	8.0	2.1	8.0	2.0
Anti-PD-L1								
HAWK, 2019	Durvalumab	16.2	22.5	6.1 (range, 0.2-24.3)	7.1	2.1	10.3	2.0
CONDOR, 2019	Durvalumab	9.0	14.9	6.0 (range, 0.3-18.0)	6.0	1.9	not reached	4.1
NCT01375842, 2018	Atezolizumab	21.9	40.6	NA^b^	6.0	2.6	7.4	NA

PD-1, Programmed death 1; PD-L1, Programmed death-ligand 1; Niv, Nivolumab group; IC, investigator’s choice group; NA, not available; ORR, objective response rate; DCR, disease control rate; OS, overall survival; PFS, progression-free survival; IQR, interquartile range.

a Patients in this group are treated with standard single agent of investigator’s choice, such as methotrexate, docetaxel or cetuximab.

b The follow-up time of 2-year update of Checkmate141 and NCT01375842 is of a minimum of 24.2 and 14 months, respectively.

### Higher Objective Response Rate in HPV Positive HNSCC Patients

We conducted a pooled analysis to assess the clinical efficacy of anti-PD-1/PD-L1 agents in HNSCC patients grouped by agents and HPV status.

**Figure 2:**
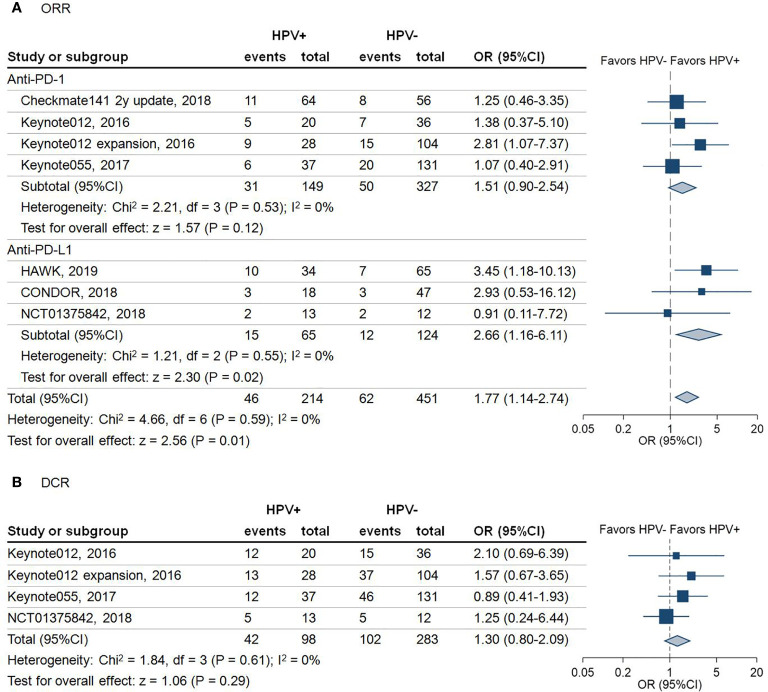
Forest plot for the efficacy of anti-programmed death-1/programmed death-ligand 1 agents in patients with different human papillomavirus status. **(A)** Objective response rate (ORR) of the included studies stratified by the type of immune checkpoint blockade. Squares indicate adjusted effect size (odds ratio [OR]). Horizontal lines represent 95% confidence interval (95%CI). Diamonds represent the pooled ORs; **(B)** Disease control rate (DCR) of the included studies. Squares indicate adjusted effect size (odds ratio [OR]). Horizontal lines represent 95%CI. Diamonds represent the pooled ORs. Pooled ORs were calculated using the fixed-effect model. PD-1, Programmed death 1; PD-L1, Programmed death-ligand 1; HPV, human papillomavirus; +, positive; -, negative.

A total of 665 patients from seven studies with a reported ORR were included in this analysis. As shown in **Figure 2A**, the results revealed that HPV positive patients had a higher ORR than HPV negative patients, regardless of anti-PD-1 or anti-PD-L1 treatment (ORR: 21.5% vs 13.7%, odds ratio (OR) = 1.77, 95% confidence interval (95%CI) = 1.14-2.74; *P* = 0.01). Subgroup analysis demonstrated that the pooled OR with use of anti-PD-1 agents was 1.51 (95%CI = 0.90–2.54; *P* = 0.12). In comparison, the pooled OR with use of anti-PD-L1 agents was 2.66 (95%CI = 1.16–6.11; *P* = 0.02) (**Figure 2A**).

### Favorable Overall Survival in HPV Positive HNSCC Patients

**Figure 3:**
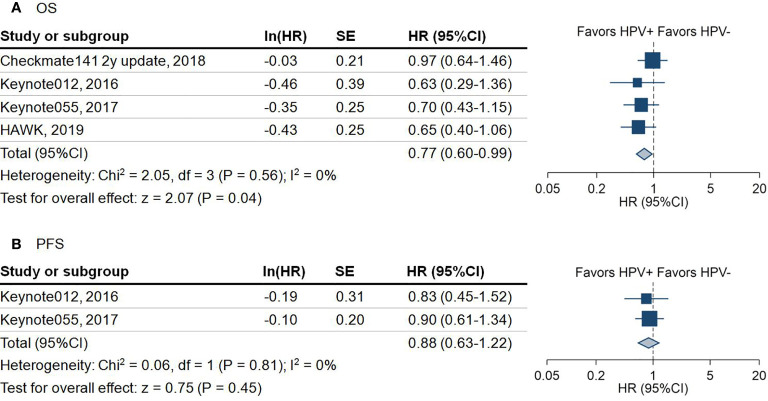
Forest plot for the survival outcome of anti-programmed death-1/programmed death-ligand 1 agents in patients with different human papillomavirus status. **(A)** Overall survival (OS) of included studies. Squares indicate adjusted effect size (hazard ratio [HR]). Horizontal lines represent 95% confidence interval (95%CI). Diamonds represent the pooled HRs. **(B)** Progression-free survival (PFS) of included studies. Squares indicate adjusted effect size (hazard ratio [HR]). Horizontal lines represent 95%CI. Diamonds represent the pooled HRs. Pooled HRs were calculated using the fixed-effect model. PD-1, Programmed death 1; PD-L1, Programmed death-ligand 1; HPV, human papillomavirus; +, positive; -, negative; SE, standard error.

447 patients available from four studies showed that HPV positive patients had significantly better overall survival than HPV negative patients (hazard ratio (HR) = 0.77; 95%CI = 0.60–0.99; *P* = 0.04) (**Figure 3A**). Because the overall survival (OS) data stratified by HPV status was not provided in the original trials, we were not capable of performing subgroup analysis of OS by anti-PD-1 and anti-PD-L1 separately.

Due to not all the trials reporting disease control rate (DCR) and progression-free survival (PFS), there were only 381 patients from four trials involved in the analysis of DCR. The pooled analysis demonstrated that HPV positive patients are 1.3 times more likely to achieve DCR compared with HPV negative patients (DCR: 42.9% vs 36.0%, OR = 1.30, 95%CI = 0.80–2.09; *P* = 0.29) (**Figure 2B**). However, this outcome did not achieve statistical significance. A similar trend of PFS in HPV positive over HPV negative status was noticed in 224 HNSCC patients (HR = 0.88; 95%CI = 0.63–1.22; *P* = 0.45) (**Figure 3B**).

## Discussion

The impact of immunotherapy in the treatment of head and neck squamous cell carcinoma (HNSCC) has been rapidly progressing, with an associated survival benefit in approximately 20-30% of patients (31, 34, 35). We hypothesized that the distinct immunological tumor landscapes of HPV positive and negative HNSCC patients might confer a difference in survival rates and tumor response after anti-PD-1/PD-L1 therapy (36). Previous evidence suggests that HPV may promote the expression of PD-L1 and PD-1 mediated through an IFN-γ related response (37, 38). Consequently, this may support the hypothesis that HPV is a favorable factor in both anti-PD-1 and anti-PD-L1 treated cancer patients. Through analysis of seven studies, including 814 patients, we demonstrated that HPV positive HNSCC patients treated with anti-PD-1 or anti-PD-L1 agents displayed significantly longer OS than HPV negative HNSCC patients, which is in concordance with a similar association observed in HPV positive vs. negative patients undergoing surgery or chemoradiotherapy (17, 18). While the difference in PFS and DCR was not significant between HPV positive and negative patients, the limited number of studies here may be a potential factor influencing this result. Specifically, there were only four studies that reported DCR and two studies that reported PFS. As more trial data emerge with longer follow-up time, the true association of HPV status with DCR and PFS will be more definitively ascertained.

To our knowledge, this is the first study to report an association with improved outcomes using anti-PD-L1 agents in HPV positive HNSCC patients. The explanation for this observed association is likely complicated and multifactorial. In addition to binding to PD-1, PD-L1 may inhibit T cell proliferation and induce immune tolerance *in vivo* and *in vitro via* the interaction with other receptors such as CD80 (39). Blocking PD-L1 on dendritic cells (DC) relieves cis sequestration of CD80, which allows CD80/CD28 interaction to enhance T cell priming (40). We have previously shown that HPV positive HNSCC patients might have a higher proportion of DCs than HPV negative patients (41). Furthermore, it has been shown that the HPV16 E7 oncoprotein may promote increased CD80 expression on DCs (42). Therefore, combined blockade of PD-1/PD-L1 and CD80/PD-L1 interactions by anti-PD-L1 agents in HPV positive HNSCC patients may represent a possible mechanism for increased benefit in these patients. Additionally, HPV16 E6/E7 oncoprotein may promote Akt activity (43) and glucose consumption (44). *In vitro* culture of tumor cell lines with anti-PD-L1 directly might decrease AKT phosphorylation and glucose uptake in the absence of PD-1-expressing T cells (45), which may directly restrain tumor cell growth in turn. This could be another potential mechanism of increased benefit from anti-PD-L1 agents in HPV positive HNSCC patients. While these pathways provide a rational hypothesis towards explaining the difference in outcomes seen in our analysis, this does not imply that the mode of action of anti-PD-1/anti-PD-L1 inhibitors is different in HPV positive HNSCC patients compared with HPV negative. Rather, this demonstration in outcome difference suggests a need for translational research to better elucidate the underlying mechanism.

We noticed that there was a higher ORR in HPV positive over HPV negative HNSCC patients undergoing anti-PD-1 therapy in our analysis; however, this difference failed to reach significance (*P* = 0.12). As more and higher-quality trial data emerges, this difference may also trend toward significance. Importantly, the number of patients in our anti-PD-1 treated studies (476 patients) outnumber those of the anti-PD-L1 studies (189 patients) in our analysis, suggesting that the preferential effect of anti-PD-L1 therapy cannot merely be explained secondary to low numbers in the anti-PD-1 treated studies. Furthermore, the proportion of HPV positive patients in the two subgroups of anti-PD-1 and anti-PD-L1 studies is similar (31.3% vs. 34.4%, **Figure 2A**), underlining the evenness of HPV positive patients across the two different treatment-subgroups. From an immune perspective, it has been theorized in previous studies that anti-PD-1 agents would have a more extensive effect beyond the PD-L1 pathway (46), with the notion that blocking the PD-1 receptor would interfere with interactions with multiple ligands, including PD-L1 and PD-L2 (47). Here, it is important to note that PD-L2 expression is variable, and it is not as highly expressed as PD-L1 on tumor and immune cells, making its role in anti-cancer immune suppression unclear (48, 49). Differences in the expression of PD-L2 between HPV-positive and HPV-negative tumors may offer a potential explanation for the differences observed in our study.

Previous work has been published that investigates the relationship between HPV status and response to anti-PD-1/PD-L1 therapy (24, 25, 50). Patel et al. (24) and Wang et al. (25) both showed a trend towards significance for higher response rates in HPV positive vs. HPV negative tumors in patients receiving anti-PD-1/PD-L1 therapy. Additionally, a recent report indicated that immune checkpoint blockade immunotherapy enhances ORR in HPV positive HNSCC patients compared with HPV negative patients (50). However, a major limitation of this report is that it only included four clinical trials, which does not encompass the full scope of the present literature on the topic. Furthermore, the key limitation in these previously published studies is the lack of stratification by anti-PD-1 or anti-PD-L1 therapy separately. Our study is the first to do so, and this has demonstrated the possibility of a meaningful difference in outcome for HPV positive HNSCC patients treated with either anti-PD-1 or anti-PD-L1 blockers. In our study, we used restricted inclusion criteria to ensure the quality of all the eligible studies (Keynote-040 (34) and Keynote-048 (35) were excluded due to no published ORR for HPV positive patients, and other data from trials like EAGLE (NCT02369874), NCT02684253 (51) were not available yet), but were still able to include a larger number of patients with HPV information.

### Limitations

Our study has several limitations. Firstly, some information was not available in all included studies, which prevented us from performing other informative sub-analyses such as stratification by anatomic subsite, the combination of PD-L1 expression and HPV subgroups, and drug dosage. In further studies, anatomic subsite in the head and neck is an essential factor to consider as it is known that the prognostic value of HPV status, based on available data, is thus far limited to the oropharynx (52). Particularly, as PFS is really the gold standard for the effect of therapy, the limited data of PFS restrict the value of our conclusion. Additionally, as there are no HPV diagnostic tests with FDA regulatory approval for head and neck cancers, the methodology to determine HPV status across the included trials differed based on the local institution or licensed lab. The most common method in the included trials was p16 immunohistological staining applied to oropharyngeal cancer as well as non-oropharyngeal cancer, which may be imperfect and would therefore bring bias, albeit minor, to the analysis (**Table 1**). Furthermore, a vast majority of HPV positive tumors are located within the oropharynx. The overall PD-L1 expression information is summarized in **Supplementary Table 3**. Nevertheless, the interaction between PD-L1 and HPV is difficult to estimate due to the currently available data. It is possible that elements of our analysis are confounded by differences in response and survival related to the subsite and PD-L1 expression itself. These considerations should be addressed carefully as more randomized controlled trial data matures in order to confirm our findings and to explore other possible factors related to response to immunotherapy in HNSCC.

## Conclusion

HPV positive HNSCC patients display improved outcomes with PD-1/PD-L1 axis blockade as compared to HPV negative HNSCC patients. These improved outcomes are likely driven to a greater extent by anti-PD-L1 inhibitors. However, randomized controlled trials with greater numbers of patients are needed for validation of these early findings.

## Data Availability Statement

The original contributions presented in the study are included in the article/**Supplementary Material**. Further inquiries can be directed to the corresponding authors.

## Author Contributions

GZ, YL, RM, and XZ conceived and designed the study. YX, DH, and IW extracted, analyzed, and interpreted the data. YX and GZ drafted the article. TS, CM, YL, RM, and XZ did the critical revision of the article for important intellectual content. All authors contributed to the article and approved the submitted version.

## Funding

This research was supported by the National Key Research and Development Project of China (Nos. 2020YFC1316900 and 2020YFC1316901), the Project of Hunan Health Commission (B2019165), National Natural Science Foundation of China (Nos. 82073009, 81974424, 81874133, 81773243, 81772903, and 81602389), Natural Science Foundation of Hunan Province (Nos. 2020JJ4827, 2019JJ40481, and 2019JJ50944) and the Huxiang Young Talent Project (No. 2018RS3024).

## Conflict of Interest

The authors declare that the research was conducted in the absence of any commercial or financial relationships that could be construed as a potential conflict of interest.
