# Supplementary material for: Programmed Death-1/Programmed Death-Ligand 1-Axis Blockade in Recurrent or Metastatic Head and Neck Squamous Cell Carcinoma Stratified by Human Papillomavirus Status: A Systematic Review and Meta-Analysis
Source: Front Immunol. 2021 Apr 7;12:645170. doi: 10.3389/fimmu.2021.645170 (PMC8058384; doi:10.3389/fimmu.2021.645170)
Supplement: Supplementary file 1 [file DataSheet_1.docx]

***Supplementary Material***

1. **Supplementary Data**
2. **Search Strategies**

**PubMed (February 28, 2021) : 592results**

(HPV or Human papillomavirus) and (Immunotherapy or nivolumab or pembrolizumab or cemiplimab or atezolizumab or durvalumab or avelumab or PD-1 or PD-L1 or PD1 or PDL1 or checkpoint) and (head and neck or head and neck cancer or head and neck neoplasm or head and neck tumor or head and neck carcinoma or HNC or HNSCC or SCCHN)

**EMBASE (February 28, 2021) : 657results**

(‘HPV’:ab,ti or ‘Human papillomavirus’:ab,ti) and (‘Immunotherapy’:ab,ti or ‘nivolumab’:ab,ti or ‘pembrolizumab’:ab,ti or ‘cemiplimab’:ab,ti or ‘atezolizumab’:ab,ti or ‘durvalumab’:ab,ti or ‘avelumab’:ab,ti or ‘PD-1’:ab,ti or ‘PD-L1’:ab,ti or ‘PD1’:ab,ti or ‘PDL1’:ab,ti or ‘checkpoint’:ab,ti) and (‘head and neck’:ab,ti or ‘head and neck cancer’:ab,ti or ‘head and neck neoplasm’:ab,ti or ‘head and neck tumor’:ab,ti or ‘head and neck carcinoma’:ab,ti or ‘HNC’:ab,ti or ‘HNSCC’:ab,ti or ‘SCCHN’:ab,ti)

**Cochrane Library (February 28, 2021) : 95results**

(HPV or Human papillomavirus) and (Immunotherapy or nivolumab or pembrolizumab or cemiplimab or atezolizumab or durvalumab or avelumab or PD-1 or PD-L1 or PD1 or PDL1 or checkpoint) and (head and neck or head and neck cancer or head and neck neoplasm or head and neck tumor or head and neck carcinoma or HNC or HNSCC or SCCHN)

**Web of Science (February 28, 2021) : 742results**

**2 Supplementary Figures and Tables**

**2.1 Supplementary Figures**

**2.1.1 Supplementary Figure 1. Risk of bias summary**

**Abbreviations: +, low risk; ?, unclear risk.**

| **2.2 Supplementary Tables**  **2.2.1 Supplementary Table 1. Quality assessment of single-arm studies^a^ according to the Newcastle-Ottawa scale** | | | | | | | | | | |
| --- | --- | --- | --- | --- | --- | --- | --- | --- | --- | --- |
| **Study** | **Selection** | | | | **Comparability** | **Exposure** | | | **Total score** | **Risk of bias** |
|  | **S1** | **S2** | **S3** | **S4** | **C1** | **E1** | **E2** | **E3** | **(Max. of 8)** |  |
| Keynote012, 2016 | 1 | 0 | 1 | 0 | 0 | 1 | 1 | 1 | 5 | High |
| Keynote012 expansion, 2016 | 1 | 0 | 1 | 0 | 0 | 1 | 1 | 1 | 5 | High |
| Keynote055, 2017 | 1 | 0 | 1 | 0 | 0 | 1 | 1 | 1 | 5 | High |
| HAWK, 2019 | 1 | 0 | 1 | 0 | 0 | 1 | 1 | 1 | 5 | High |
| NCT01375842, 2018 | 1 | 0 | 1 | 0 | 0 | 1 | 1 | 1 | 5 | High |
| ^a^The studies are scored on the scale of cohort studies of the Newcastle-Ottawa Assessment Scale. | | | | | | | | | | |

| **2.2.2 Supplementary Table 2. The overview of anatomical subsites in the study** | | | | | | | | | |
| --- | --- | --- | --- | --- | --- | --- | --- | --- | --- |
| **Study** | **N** | **Oral cavity**  **(%)** | **Nasal cavity**  **(%)** | **Larynx**  **(%)** | **Pharynx^a^**  **(%)** | **Nasopharynx**  **(%)** | **Oropharynx**  **(%)** | **Hypopharynx**  **(%)** | **Others**  **(%)** |
| **Anti-PD-1** |  |  |  |  |  |  |  |  |  |
| Checkmate141 2y update, 2018 | 240 | 108(45) | NA | 34(14) | 92(38) | NA | NA | NA | 6(3) |
| Keynote012, 2016 | 60 | 11(18) | 4(7) | 2(3) | 20(34) | 3(5) | 16(27) | 1(2) | 23(38) |
| Keynote012 expansion, 2016 | 132 | 17(13) | 8(6) | 16(12) | 77(58) | 5(4) | 60(45) | 12(9) | 14(11) |
| Keynote055, 2017 | 171 | 28(16) | 1(1) | 30(18) | 108(63)^b^ | NA | 100(58) | 7(4) | 4(2) |
| **Anti-PD-L1** |  |  |  |  |  |  |  |  |  |
| HAWK, 2019 | 112 | 47(42) | NA | 15(13) | 49(44) | NA | 40(36) | 9(8) | 1(1) |
| CONDOR, 2018 | 67 | 15(22) | NA | 17(25) | 33(49) | NA | 25(37) | 8(12) | 2(3) |
| NCT01375842, 2018 | 32 | 7(22) | NA | 2(6) | 23(72) | 4(13) | 18(56) | 1(3) | NA |
| Abbreviations: N, number of patients; PD-1, programmed death 1; PD-L1, programmed death-ligand 1; NA, not available. ^a^The value of pharynx is the sum of the number of nasopharynx, oropharynx and hypopharynx.  ^b^In the Keynote055 study, the number of patients with cancer located in pharynx is 1, so we view the total number of pharynx in this trial as 108. | | | | | | | | | |

| **2.2.3 Supplementary Table 3. The overview of PD-L1 expression status and its influence on clinical efficacy** | | | | |
| --- | --- | --- | --- | --- |
| **Study** | **PD-L1 expression cutoff** | **events** | **total** | **ORR (%)** |
| **Anti-PD-1** |  |  |  |  |
| Checkmate141 2y update | ≥1% tumor | 17 | 96 | 17.7 |
|  | <1% tumor | 9 | 76 | 11.8 |
| Keynote012 | ≥1% tumor cells or stroma | 12 | 56 | 21.4 |
| Keynote012 expansion | ≥1% tumor and immune cells | 23 | 107 | 21.5 |
|  | <1% tumor and immune cells | 1 | 25 | 4.0 |
|  | ≥1% tumor cells | 17 | 89 | 19.1 |
|  | <1% tumor cells | 7 | 43 | 16.3 |
| Keynote055 | ≥1% CPS (tumor and mononuclear inflammatory cells) | 25 | 140 | 17.9 |
|  | <1% CPS | 3 | 26 | 11.5 |
|  | ≥50% CPS | 13 | 48 | 27.1 |
|  | <50% CPS | 15 | 118 | 12.7 |
| **Anti-PD-L1** |  |  |  |  |
| HAWK | ≥25% tumor cells | 17 | 99 | 17.2 |
| CONDOR | <25% tumor cells | 6 | 65 | 9.2 |
| NCT01375842 | ≥5% tumor-infiltrating immune cells | 4 | 15 | 26.7 |
| Abbreviations: PD-L1, programmed death-ligand 1; PD-1, programmed death 1; ORR, objective response rate; CPS, combined positive score. | | | | |
